# Metagenomic analysis of basal ice from an Alaskan glacier

**DOI:** 10.1186/s40168-018-0505-5

**Published:** 2018-07-05

**Authors:** Masood ur Rehman Kayani, Shawn M. Doyle, Naseer Sangwan, Guanqun Wang, Jack A. Gilbert, Brent C. Christner, Ting F. Zhu

**Affiliations:** 1 School of Life Sciences, Tsinghua-Peking Joint Center for Life Sciences, Center for Synthetic and Systems Biology, Ministry of Education Key Laboratory of Bioinformatics, Tsinghua University, Beijing, 100084 China; 2 College of Geosciences, Texas A&M University, College Station, TX 77843 USA; 3 Biosciences Division (BIO), Argonne National Laboratory, 9700 South Cass Avenue, Argonne, IL 60439 USA; 4 Department of Surgery, The Microbiome Center, University of Chicago, 5841 South Maryland Avenue, MC 5029, Chicago, IL 60637 USA; 5 The Microbiome Center, Marine Biological Laboratory, 7 MBL Street, Woods Hole, MA 02543 USA; 6 Department of Microbiology and Cell Science, Biodiversity Institute, University of Florida, Gainesville, FL 32611 USA

**Keywords:** Microbiome, Metagenomics, Glacier, Basal ice layer

## Abstract

**Background:**

Glaciers cover ~ 10% of land but are among the least explored environments on Earth. The basal portion of glaciers often harbors unique aquatic microbial ecosystems in the absence of sunlight, and knowledge on the microbial community structures and their metabolic potential is very limited. Here, we provide insights into the microbial lifestyle present at the base of the Matanuska Glacier, Alaska.

**Results:**

DNA and RNA were extracted from samples of the Matanuska Glacier basal ice. Using Illumina MiSeq and HiSeq sequencing, we investigated the microbial diversity with the metagenomic shotgun reads and 16S ribosomal RNA data. We further assembled 9 partial and draft bacterial genomes from the metagenomic assembly, and identified key metabolic pathways such as sulfur oxidation and nitrification. Collectively, our analyses suggest a prevalence of lithotrophic and heterotrophic metabolisms in the subglacial microbiome.

**Conclusion:**

Our results present the first metagenomic assembly and bacterial draft genomes for a subglacial environment. These results extend our understanding of the chemical and biological processes in subglacial environments critically influenced by global climate change.

**Electronic supplementary material:**

The online version of this article (10.1186/s40168-018-0505-5) contains supplementary material, which is available to authorized users.

## Background

Subglacial ecosystems are associated with hydrological networks at the base of glaciers and ice sheets that transport water discharged proglacially or into the ocean [1]. The role of glaciated environments in effecting the biogeochemistry of downstream ecosystems has only recently been appreciated [2]. Functional microbial ecosystems have been documented within subglacial aquatic environments in a range of alpine and valley glaciers [3], as well as beneath the Antarctic and Greenland ice sheets [4–7]. However, given the difficulty in accessing these environments, our understanding of microbial life in the ecosystems remains very limited.

Basal ice forms in the deepest portion of a glacier where the ice is in direct contact with the underlying bedrock, and its composition and structure become altered through interactions with glacier bed [8]. Basal processes such as localized pressure-induced melt-refreezing [9, 10] or glaciohydraulic supercooling [11] can capture materials (i.e., water and sediments) from the subglacial environment and transport them within the basal ice layer. Microorganisms that are associated with subglacial water and sediment become entrained in the basal ice layer together with nutrients important to supporting ecological processes within the subglacial environment [8, 12].

The Matanuska Glacier is a large, terrestrial valley glacier located in the Chugach Mountains of Southcentral Alaska, approximately 138 km north of Anchorage. It flows north ~ 45 km from the Ted Stevens Ice Field and ranges in width from ~ 3 km near the equilibrium line to ~ 5 km along its terminus [13]. A number of hydrological, geochemical, and glaciological studies have been conducted on the Matanuska Glacier [11, 14–16] partly due to observations of supercooled subglacial water which emerges through vents, conduits, and crevasses at the terminus [11, 17, 18]. This meltwater produces frazil ice that can accrete, forming a debris-laden basal zone. Sediments within the subglacial discharge and entrained in basal ice are derived from Lower Cretaceous to Upper Jurassic (flysh, greenstone, limestone, chert, granodiorite, glaucophane-bearing greenshist, and layered gabbro and serpentinite), and Cretaceous to Upper Jurassic (graywacke, slate, argillite, volcanic detritus, and interbedded mafic) rocks.

In this study, we examined the microbial community inhabiting the basal ice layer from Matanuska Glacier, Alaska, using 16S rRNA gene (rDNA), 16S rRNA transcript (rRNA), and metagenomic sequencing analysis. These data were used to assemble 9 partial and draft bacterial genomes *de novo* and to assess the metabolic activity of specific microbial taxa based on 16S rRNA to rDNA ratios.

## Methods

Samples of exposed basal ice at the terminus of the Matanuska Glacier (Additional file 1: Figure S1) were collected in July 2013 using an electric chainsaw (61.776 N 147.761 W, 510-m elevation). The glaciological and physical properties of the basal ice at the Matanuska Glacier have been described previously [11]. Following sample decontamination as described previously [12], nucleic acids were extracted (see Additional file 1: Supplementary Methods). The Illumina MiSeq platform (Illumina, CA, USA) was used to perform 16S rRNA/rDNA sequencing with paired-end read length of 250 bp (see Additional file 1: Supplementary Methods). Sequences were clustered as operational taxonomic units (OTU) at 3% dissimilarity using the furthest neighbor algorithm and classified using a naïve Bayesian classifier and the Ribosomal Database Project training set (Release 9). Representative sequences for each OTU were taxonomically classified using Greengenes (v123), SILVA (v13_5), and NCBI GenBank (Release 221). The metagenomic shotgun library was sequenced using an Illumina HiSeq 2500 system and read length of 150 bp. To assess taxonomic diversity of the metagenome, the paired-end reads were analyzed using Phylosift v1.0.1. The draft genomes were obtained using MetaBAT v2.12 after assembling the raw reads using the Iterative De-Bruijn Graph de novo assembler (IDBA-UD). Their completeness levels were assessed using CheckM (v0.9.7) while the functional annotations were performed using rapid annotation using subsystem technology (RAST) and KEGG automatic annotation server (KAAS) servers (see Additional file 1: Supplementary Methods).

### Initial findings

**Fig. 1:**
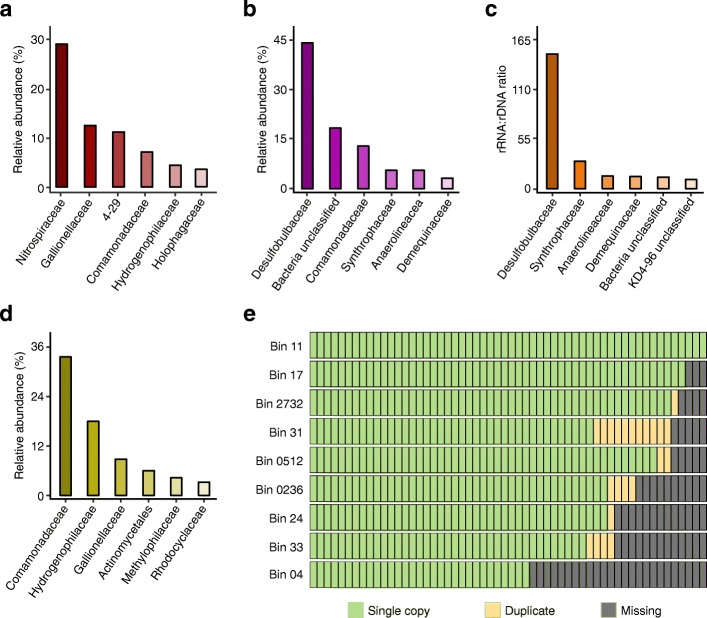
Taxonomic classification, abundance, and genome binning of the Matanuska basal ice layer metagenome. Relative abundance of top bacterial families identified by 16S rDNA (**a**) and rRNA (**b**) sequencing data. **c** Top potential metabolically active bacterial OTUs inferred by rRNA to rDNA ratio. **d** Relative abundance of top bacterial families identified by metagenomic data. **e** Partial and draft genome quality assessment plot indicating completeness and contamination levels of each GB

The 16S rRNA and rDNA sequencing data revealed 2122 curated OTUs, with low microbial diversity (Inverse Simpson = 20.2 and 4.2, respectively) (Additional file 1: Table S1) and high Good’s coverage values (≥ 99.8%). Based on the rDNA sequencing data, the microbial community was dominated by bacteria (2091 OTUs) that included families such as *Nitrospiraceae* and *Gallionellaceae* (Fig. 1a). In contrast, the *Desulfobulbaceae* and *Comamonadaceae* families were of the highest abundance based on the 16S rRNA sequencing data (Fig. 1b). To identify microbial community members that could be metabolically active [19], ratios of 16S rRNA:rDNA relative abundance were calculated, the results of which showed that six OTUs had rRNA to rDNA ratios > 5, including members of the *Desulfobulbaceae*, *Syntrophaceae* (Fig. 1c, Additional file 1: Table S2 and Additional file 2: Table S3).

The metagenomic sequencing data produced taxonomic classification results similar to the 16S rRNA data, with bacteria dominating the microbial community and high abundances of *Comamonadaceae*, *Hydrogenophilaceae*, and other families (Fig. 1d). Methanogens have been documented previously in subglacial environments [5, 8]; however, archaeal sequences were of low abundances in our basal ice metagenomic data (0.2%), and ~ 1.5% of the OTUs in 16S rDNA library were classified as archaea.

Assembly of the metagenomic sequencing data allowed the recovery of 9 partial and draft genomes (further referred as Genome Bins (GBs)) with a completion level ≥ 70% and contamination level ≤ 10% (Fig. 1e, Additional file 2: Tables S4–S5). These GBs were classified within the genera *Anaerolinea* (Bin 11 and Bin 33), *Synthrophus* (Bin 2732 and Bin 31), and *Thiobacillus* (Bin 04), whereas the other 4 (Bin 0236, Bin 0512, Bin 17, and Bin 24) were unclassifiable and only identified as bacteria. The genomic statistics (genome lengths, contigs, genes, N50, and %GC, etc.) for the 9 GBs are summarized in Additional file 1: Figures S2-S3 and Additional file 2: Table S6.

In the absence of light, chemolithotrophy may support biogeochemical processes in the subglacial environment [6, 20–22]. Analyte measurement from the Matanuska basal ice sample indicated high concentration of SO_4_ (4600 ppb) and relatively lower NO_3_ concentration (60 ppb). These measurements suggested that sulfate and nitrate reduction could also be prevalent in the basal ice of Matanuska Glacier. Functional analysis of the GBs identified components of lithotrophic pathways, but likely due to incompleteness, intact pathways within a single draft genome were not recovered. Dissimilatory adenylylsulfate reductases (*aprA* and *aprB*) and sulfur oxidizing (*soxB*) genes were present in Bin 04 (Table 1). The identification of the *soxB* gene suggested potential for lithotrophic metabolism via oxidation of reduced sulfur compounds (e.g., hydrogen sulfide, sulfur, sulfite, and thiosulfate) for energy production. Furthermore, Bin 04 also possessed genes of dissimilatory nitrate reductases (*narGHI* and *napA*) and nitrite oxidoreductase (*nxrA*), implying the capability to utilize nitrate as terminal electron acceptor in anaerobic respiration. The presence or absence of key pathways and genes in the Matanuska basal ice layer GBs are summarized in Additional file 1: Figure S4 and Additional file 2: Tables S7–S9.

**Table 1:** Presence (+) and absence (−) of key predicted genes in the 9 GBs

Category	Subcategory	Predicted gene	Bin 04	Bin 11	Bin 17	Bin 24	Bin 31	Bin 33	Bin 0236	Bin 0512	Bin 2732
Nitrogen metabolism	Dissimilatory nitrate reduction	Nitrate reductase (*narGHI*)	+	−	−	−	−	−	−	−	−
		Periplasmic nitrate reductase	+	−	−	−	−	−	−	−	−
		Nitrite reductase (*nirD*)	−	−	−	−	−	−	+	−	−
		Nitrite reductase (*nrfA*)	−	+	−	−	−	−	−	−	−
	Denitrification	Periplasmic nitrate reductase	+	−	−	−	−	−	−	−	−
	Nitrification	Nitrite oxidoreductase subunit (*nxrA*)	+	−	−	−	−	−	−	−	−
	Nitrogen fixation	Nitrogenase iron proteins (*nifDKH*)	−	−	+	−	−	−	−	−	−
Sulfur metabolism	Dissimilatory sulfate reduction	Adenylylsulfate reductase subunit A (*aprA*)	+	−	+	−	−	−	−	−	−
		Adenylylsulfate reductase subunit B (*aprB*)	+	−	−	−	−	−	−	−	−
	SOX system	Sulfur oxidizing protein (*soxB*)	+	−	−	−	−	−	−	−	−
Carbon metabolism	CO_2_ Fixation	Ribulose bisphosphate carboxylase (small chain)	+	−	−	−	−	−	−	−	+
		Ribulose bisphosphate carboxylase (large chain)	+	−	−	−	−	−	−	−	+
		RuBisCO activation protein (*cbbO*)	+	−	−	−	−	−	−	−	−
		RuBisCO activation protein (*cbbQ*)	+	−	−	−	−	−	−	−	−
		RuBisCO transcriptional regulator (*cbbR*)	+	−	−	−	−	−	−	−	+
Respiration	Complex I	NADH Ubiquinone oxidoreductase	+	+	−	−	−	+	−	−	+
	Complex II	Succinate dehydrogenase	+	+	−	−	+	+	−	−	+

Genes of carbon metabolism pathways in the GBs were mostly involved in central carbohydrate metabolisms such as glycolysis, gluconeogenesis, and pyruvate metabolism (Additional file 2: Table S9). However, CO_2_ fixation enzymes, especially Ribulose bisphosphate carboxylase (RuBisCO), were also identified in Bin 04. The ability to perform CO_2_ fixation supports the hypothesis for microbial lithoautotrophic metabolism via sulfur oxidation. The GBs also contained genes related to cold and oxidative stress tolerance: Bin 11 and Bin 31 contained genes of cold shock protein (*cspA*), whereas Bin 04, Bin 0236, and Bin 2732 possessed genes of catalase and superoxide dismutase.

In summary, our 16S rRNA/rDNA and metagenomic sequencing data suggested the presence of abundant bacterial taxa within the Matanuska basal ice layer, and functional predictions suggested the presence of genes involved in sulfur lithotrophy as well as nitrate/sulfate respiration.

### Future directions

The current data presented a metagenomic shotgun analysis of a basal ice layer of glacier. The metagenomic assembly and draft genomes are, to the best of our knowledge, the only reference datasets currently available for a subglacial ecosystem, providing valuable insight into the biogeochemical potential of the microbial communities within these permanently frozen ecosystems. The inaccessibility of these environments remains a major hindrance, and a limitation of this study is that it represents a single basal ice cryofacies (i.e., dispersed ice) from a single glacier. More thorough sampling and analysis of multiple basal ice cryofacies (e.g., clean, banded, solid, and stratified) and different glacier types (i.e., cold, temperate, and polythermal) are necessary to establish a comprehensive understanding of the diversity and function of microbial communities inhabiting the basal zones of glaciers. Despite the limitations, our results may be used in several downstream studies such as comparative analysis of the basal ice zones and other glacial environments. The draft genomes obtained by this study may also be important for comparative genomics and evolutionary studies.

## Additional files

Additional file 1: Supplementary Information. (PDF 1581 kb)

Additional file 2: Supplementary Tables. (XLSX 142 kb)
