# Supplementary material for: Metagenomic analysis of basal ice from an Alaskan glacier
Source: Microbiome. 2018 Jul 5;6:123. doi: 10.1186/s40168-018-0505-5 (PMC6034282; doi:10.1186/s40168-018-0505-5)
Supplement: Supplementary file 1 — Supplementary Information. (PDF 1581 kb) [file 40168_2018_505_MOESM1_ESM.pdf]

Additional File 1

Metagenomic analysis of basal ice from an Alaskan glacier

Masood ur Rehman Kayani[1#], Shawn M. Doyle[2#], Naseer Sangwan[3,4], Guanqun Wang[1], Jack A. Gilbert[3,4,5]*, Brent C. Christner[6]*, Ting F. Zhu[1]*

[1]School of Life Sciences, Tsinghua-Peking Joint Center for Life Sciences, Center for Synthetic and Systems Biology, Ministry of Education Key Laboratory of Bioinformatics, Tsinghua University, Beijing 100084, China

[2]College of Geosciences, Texas A&M University, College Station, TX 77843, USA

[3]Biosciences Division (BIO), Argonne National Laboratory, 9700 South Cass Avenue, Argonne, IL 60439, USA

[4]Department of Surgery, University of Chicago, 5841 South Maryland Avenue, MC 5029, Chicago, IL 60637, USA

[5]Marine Biological Laboratory, 7 MBL Street, Woods Hole, MA 02543, USA

[6]Department of Microbiology and Cell Science, Biodiversity Institute, University of Florida, Gainesville, FL 32611, USA

[#]These authors contributed equally

*To whom correspondence should be addressed: E-mail: gilbertjack@uchicago.edu (J.A.G.); xner@ufl.edu (B.C.C.); tzhu@tsinghua.edu.cn (T.F.Z.), ORCID: 0000-0003-0897-0303

Table of Contents Page

Supplementary Methods 2

Supplementary Figures S1-S4 5

Supplementary Tables S1-S2 9

Supplementary References 11

Supplementary Tables S3-S9 Additional File 2

Supplementary Methods

Sample collection and decontamination

Samples of basal ice at the glacier terminus were collected in July, 2013 from the Matanuska Glacier (elevation 510 m) using an electric chainsaw with a carbide-tipped chain (06V 0459835 6849428). The ice samples were shipped frozen to Louisiana State University and stored at -20 ^{o}C. Samples were decontaminated using a modified version of the decontamination protocol described previously [1]. Briefly, the outer contaminated surface was physically removed via scraping with a sterilized stainless steel microtome blade in a class 100 laminar flow hood. After physical removal of the outermost surface, the freshly exposed ice was washed with 0.22-μm filtered 95% ethanol and rinsed with ice-cold, twice autoclaved deionized water (0.22-μm filtered). The decontamination procedure removed an annulus of approximately five millimeters from the outer surface of the samples. The cleaned ice samples were sealed within a sterile container and melted at 4 ^{o}C.

Nucleic acid extraction

DNA was extracted from 20 g (wet weight) of the basal ice-sediment slurry using PowerMax Soil DNA Isolation kit (MoBio Laboratories, CA, U.S.). To increase yield, the manufacturer's protocol was modified by combining two 10 g extractions onto a single silica spin column. RNA was extracted as previously described [2]. Briefly, coarse sediment particles were removed from ~2.85 kg of the ice-sediment slurry using low-speed centrifugation (700 ×g; 10 min, 4 °C) and the supernatant containing fine clay and silt-like particles was filtered onto 90 mm, 0.2 μm pore size Supor filter which was cut into small pieces and transferred into a 7 ml bead beating tube for nucleic acid extraction. The extracted RNA was incubated for 1 h at 37 ^{o}C with 4 U TURBO DNase from the TURBO DNA-free kit (Ambion, TX, U.S.). Subsequently, the RNA was purified with MEGAclear clean up columns (Ambion, TX, U.S.) followed by an additional ethanol precipitation step. The RNA pellet was suspended in 50 μl TE buffer and stored at -80 ^{o}C. DNA and RNA were quantified using a 20/20 n Luminometer (Turner Biosystems, CA, U.S.) equipped with a blue fluorescence module (P/N 2030-041), and the Quant-iT Picogreen assay and Ribogreen assay kits, respectively (Life Technologies, CA, U.S.).

16S rRNA sequencing and analysis

Complementary DNA (cDNA) libraries of 16S rRNA were prepared by reverse transcription of ~1 ng total RNA with SuperScript II reverse transcriptase and the 806R primer (5'-GGACTACVSGGGTATCTAAT-3') according to manufacturer's protocol. Negative controls without reverse transcriptase were included to monitor for genomic DNA contamination. The V4 (hyper-variable) region of the 16S rRNA gene was amplified from the extracted basal ice DNA and cDNA libraries using the 515F and 906R Golay barcoded and adapter ligated primers required for Illumina MiSeq sequencing [3]. The amplicons (~350 bp) were quantified fluorometrically as described above and pooled at equimolar concentrations. The pooled amplicons were purified using MoBio UltraClean PCR Cleanup kit (MoBio Laboratories, CA, U.S.) and sent to the Georgia Genomics Facility (Athens, GA, U.S.) for Illumina MiSeq sequencing (v2 chemistry, 2 × 250 bp). The raw sequencing reads were analyzed with a combination of MOTHUR v1.33.3 [4] and SINA (SILVA Incremental Aligner) v1.2.11 [5]. Sequences containing ambiguous bases or homopolymers (≥8 bp) were excluded during quality filtration and paired-end read assembly. The processed sequences were aligned using SINA to the SILVA non-redundant 16S rRNA reference database (v.119) and UCHIME was used to identify and remove chimeras [3]. Sequences were clustered as operational taxonomic units (OTU) at 3% dissimilarity using the furthest neighbor algorithm and classified using a naïve Bayesian classifier and the Ribosomal Database Project training set (Release 9). Representative sequences for each OTU were taxonomically classified using Greengenes (version 13_5), SILVA (version 123), and NCBI GenBank (Release 221). MOTHUR was also used to calculate diversity, richness, coverage, and dissimilarity indices. To infer the metabolically active OTUs, the ratios of 16S rRNA transcript (rRNA) and 16S rRNA gene (rDNA) relative abundance were calculated.

Metagenomic sequencing and analysis

The metagenomic sequencing library was constructed using 20 ng DNA and the NEBNext Ultra DNA library preparation kit (New England Biolabs, MA, U.S.). The DNA was fragmented using the Covaris 2.0 sonication system and separated on a 1.5% agarose gel. The size-selected product was end-repaired, adapter-ligated, and amplified. The amplification was limited to 12 cycles to reduce bias. The amplified library was sequenced using the Illumina HiSeq 2500 system (Illumina, CA, U.S.) with a read length of 150 bp and average insert size of 500 bp. The raw sequence data were preprocessed to remove adapter sequences and bases with quality scores ≤20. Furthermore, reads were discarded if they contained 3 or more N's or had a length <40 bp (post-Q20 trimming). To assess the taxonomic diversity of

the Matanuska Glacier basal ice layer metagenome, the paired-end reads were analyzed by using Phylosift v1.0.1 [6]. The dataset was profiled using relative abundance for bacterial, archaea, and viral taxonomic rankings.

Assembly and characterization of genome bins

For the recovery of genome bins, paired-end reads were initially assembled using IDBA-UD [7]. The range of *k*-mer was set from 20 to 100, read error repair was enabled, and contigs shorter than 200 bp were removed. The select contigs were binned into partial and draft genome bins (GBs) on the basis of their tetra-nucleotide frequencies and abundance using MetaBAT v2.12 [8]. The sequencing reads were re-mapped on the contigs using Burrows-Wheeler Aligner v0.7.3 [9], and the resulting alignment file was indexed, sorted, and processed to remove duplicates using SAMtools v1.2 [10] and used to run MetaBAT with mostly default parameters except minimum contig size for binning (-m parameter) which was modified to 1000 from its default value. The completeness and contamination levels of the GBs were assessed by CheckM v0.9.7, which uses a comprehensive catalogue of ubiquitous and single-copy genes within a phylogenetic lineage to assess the quality of microbial genomes recovered from metagenomes and other sources [11]. Marker gene compatibility and similarity of genomic characteristics among the GBs were used to merge GBs containing complementary markers and improve their quality. The partial and draft GBs with completeness and contamination levels of ≥70% and ≤10%, respectively, were assigned phylogeny using Phylosift v1.0.1 [6] and further used for gene calling using Prodigal v2.6.2 [12]. The predicted protein coding sequences were used for functional annotation. Functional annotations of the GBs were performed by rapid annotation using subsystem technology (RAST) and KEGG automatic annotation server (KAAS) servers [13, 14]. Enzymes involved in nitrogen metabolism, sulfur metabolism, carbon metabolism, and other key pathways were identified from the datasets.

Supplementary Figures

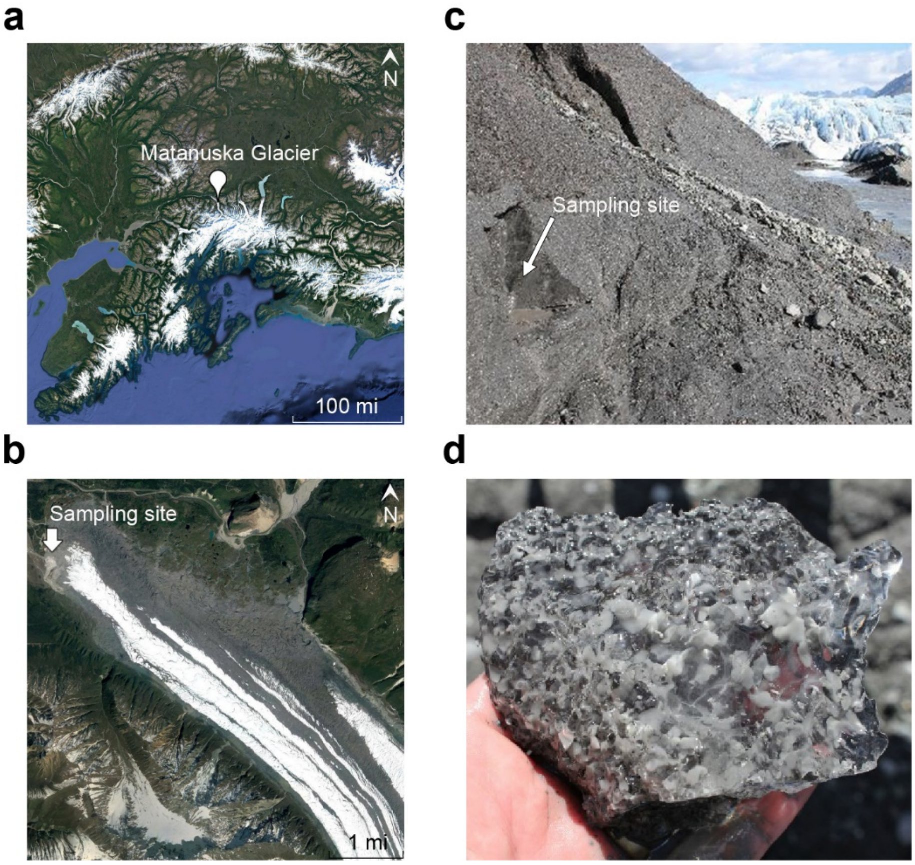

Fig. S1 Matanuska Glacier and sampling of the basal ice. **(a)** Location of Matanuska Glacier in the Alaska region (Map data © 2017 Google). **(b)** Basal ice sampling site (Imagery © 2017 DigitalGlobe, Landsat/Copernicus, Map data © 2017 Google). **(c)** Snapshot of the sampling site **(d)** Snapshot of a section of basal ice sample.

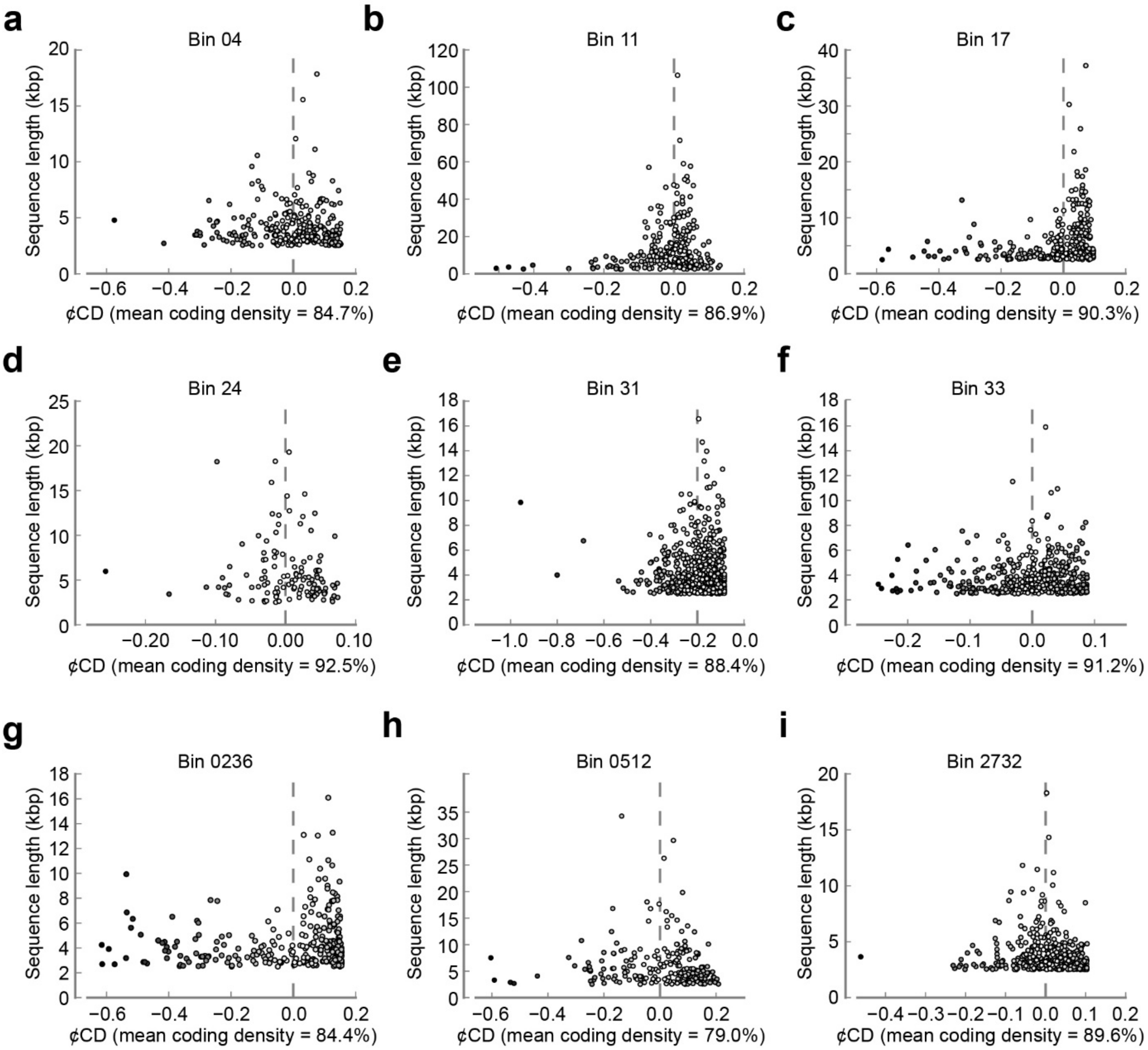

Fig. S2 Mean coding density plots for the 9 GBs.

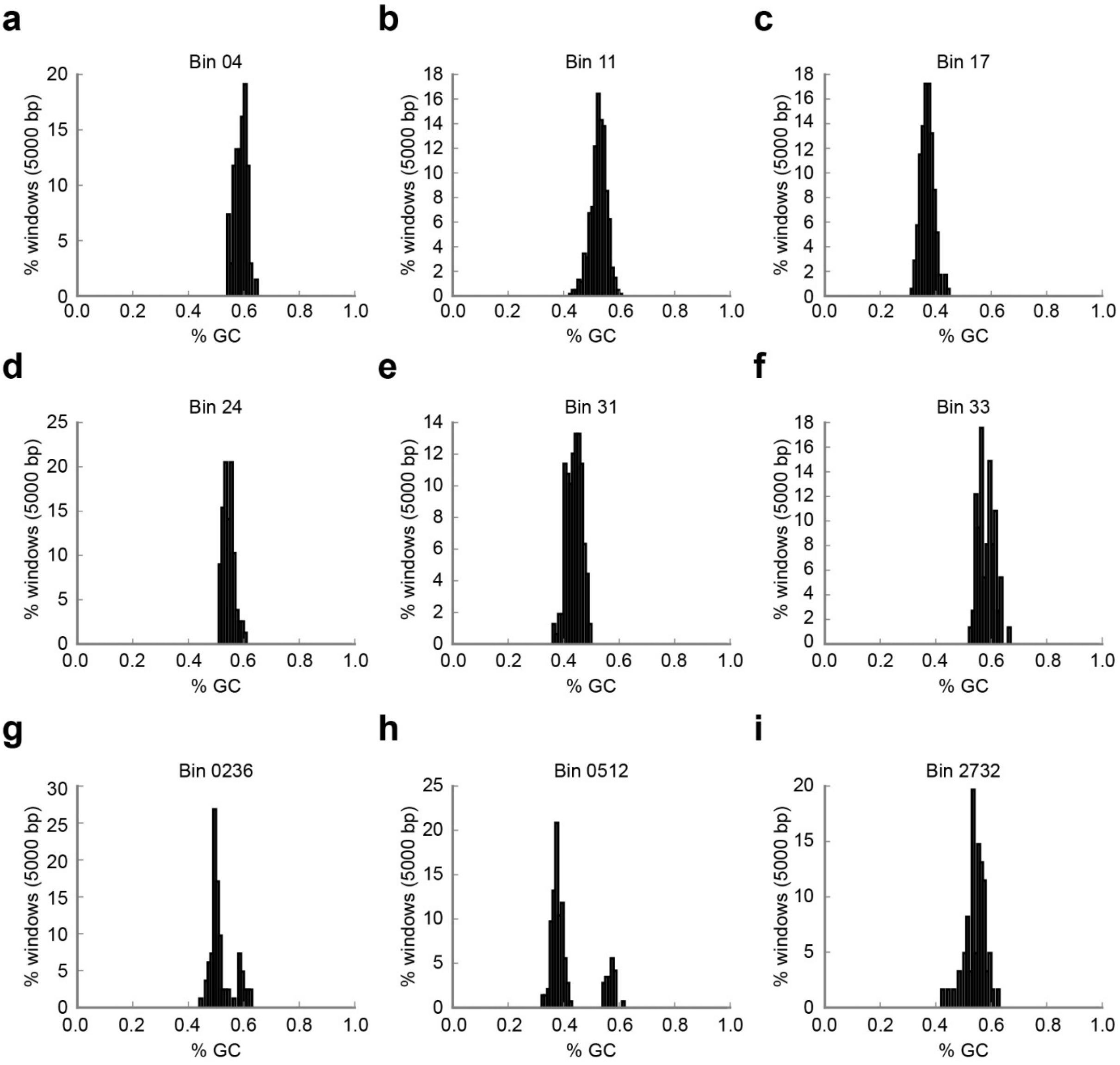

Fig. S3 Distribution of %GC content in the 9 GBs.

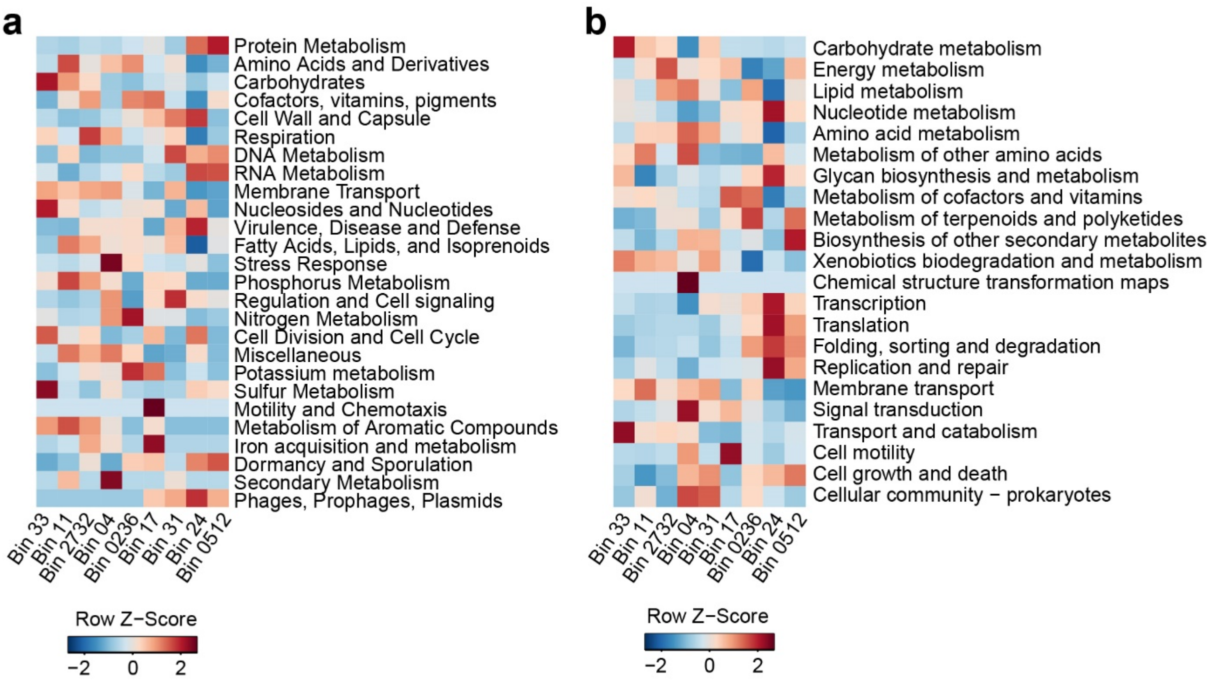

Fig. S4 Functional classification of the 9 GBs using RAST and KAAS servers. **(a)** Classification of GBs by RAST Subsystems suggests that protein, amino acid, and carbohydrate metabolism are three highly abundant subsystems. **(b)** Annotation of predicted proteins as KEGG pathways using KAAS server suggests that pathways for carbohydrate, energy, and lipid metabolism are highly abundant.

Table S1: Coverage, diversity, and richness of 16S rDNA and rRNA libraries.

Library	Reads	Good's Coverage	# of OTUs	Inverse Simpson	Chao1	ACE
rDNA	260144	0.998	1730	20.3	2145	2074
rRNA	260144	0.999	629	4.2	853	850

Table S2: Six active members of the Matanuska basal ice layer community inferred by rRNA:rDNA ratio.

OTU	rDNA relative abundance (%)	rRNA relative abundance (%)	rRNA:rDNA ratio*	Phylum	Class	Order	Family
OTU0001	0.3	44.6	151.1	Proteobacteria	Deltaproteobacteria	Desulfobacterales	Desulfobulbaceae
OTU0016	0.2	5.1	28.8	Proteobacteria	Deltaproteobacteria	Syntrophobacterales	Syntrophaceae
OTU0024	0.2	2.3	13.4	Chloroflexi	Anaerolineae	Anaerolineales	Anaerolineaceae
OTU0019	0.3	3.4	12.8	Actinobacteria	Actinobacteria	Micrococcales	Demequinaceae
OTU0008	1.2	14.1	12.1	Bacteria unclassified	Bacteria unclassified	Bacteria unclassified	Bacteria unclassified
OTU0025	0.2	2.2	9.6	Chloroflexi	KD4-96	KD4-96 unclassified	KD4-96 unclassified

*Only OTUs with rRNA abundance >2% are included.

Supplementary References

1. Christner BC, Mikucki JA, Foreman CM, Denson J, Priscu JC: Glacial ice cores: a model system for developing extraterrestrial decontamination protocols. *Icarus* 2005, 174(2):572-584.
2. Dieser M, Broemsen EL, Cameron KA, King GM, Achberger A, Choquette K, Hagedorn B, Sletten R, Junge K, Christner BC: Molecular and biogeochemical evidence for methane cycling beneath the western margin of the Greenland Ice Sheet. *ISME J* 2014, 8(11):2305-2316.
3. Edgar RC, Haas BJ, Clemente JC, Quince C, Knight R: UCHIME improves sensitivity and speed of chimera detection. *Bioinformatics* 2011, 27(16):2194-2200.
4. Schloss PD, Westcott SL, Ryabin T, Hall JR, Hartmann M, Hollister EB, Lesniewski RA, Oakley BB, Parks DH, Robinson CJ *et al*: Introducing mothur: open-source, platform-independent, community-supported software for describing and comparing microbial communities. *Appl Environ Microbiol* 2009, 75(23):7537-7541.
5. Pruesse E, Peplies J, Glockner FO: SINA: accurate high-throughput multiple sequence alignment of ribosomal RNA genes. *Bioinformatics* 2012, 28(14):1823-1829.
6. Darling AE, Jospin G, Lowe E, Matsen IV FA, Bik HM, Eisen JA: PhyloSift: phylogenetic analysis of genomes and metagenomes. *PeerJ* 2014, 2:e243.
7. Peng Y, Leung HC, Yiu S-M, Chin FY: IDBA-UD: a de novo assembler for single-cell and metagenomic sequencing data with highly uneven depth. *Bioinformatics* 2012, 28(11):1420-1428.
8. Kang DD, Froula J, Egan R, Wang Z: MetaBAT, an efficient tool for accurately reconstructing single genomes from complex microbial communities. *PeerJ* 2015, 3:e1165.
9. Li H, Durbin R: Fast and accurate short read alignment with Burrows-Wheeler transform. *Bioinformatics* 2009, 25(14):1754-1760.
10. Li H, Handsaker B, Wysoker A, Fennell T, Ruan J, Homer N, Marth G, Abecasis G, Durbin R: The Sequence Alignment/Map format and SAMtools. *Bioinformatics* 2009, 25(16):2078-2079.
11. Parks DH, Imelfort M, Skennerton CT, Hugenholtz P, Tyson GW: CheckM: assessing the quality of microbial genomes recovered from isolates, single cells, and metagenomes. *Genome Res* 2015, 25(7):1043-1055.
12. Hyatt D, Chen G-L, LoCascio PF, Land ML, Larimer FW, Hauser LJ: Prodigal: prokaryotic gene recognition and translation initiation site identification. *BMC bioinformatics* 2010, 11(1):119.
13. Aziz RK, Bartels D, Best AA, DeJongh M, Disz T, Edwards RA, Formsma K, Gerdes S, Glass EM, Kubal M *et al*: The RAST Server: rapid annotations using subsystems technology. *BMC genomics* 2008, 9:75.
14. Moriya Y, Itoh M, Okuda S, Yoshizawa AC, Kanehisa M: KAAS: an automatic genome annotation and pathway reconstruction server. *Nucleic Acids Res* 2007, 35(Web Server issue):W182-185.
